# Supplementary material for: The interactome of intact mitochondria by cross-linking mass spectrometry provides evidence for coexisting respiratory supercomplexes
Source: Mol Cell Proteomics. 2017 Dec 8;17(2):216–32. doi: 10.1074/mcp.RA117.000470 (PMC5795388; doi:10.1074/mcp.RA117.000470)
Supplement: Supplemental Data [file supp_RA117.000470_133922_0_supp_23978_fzffwf.zip › spectra_annotation/mito_spectra_annotation/132-2-4-1-15-1.pdf]

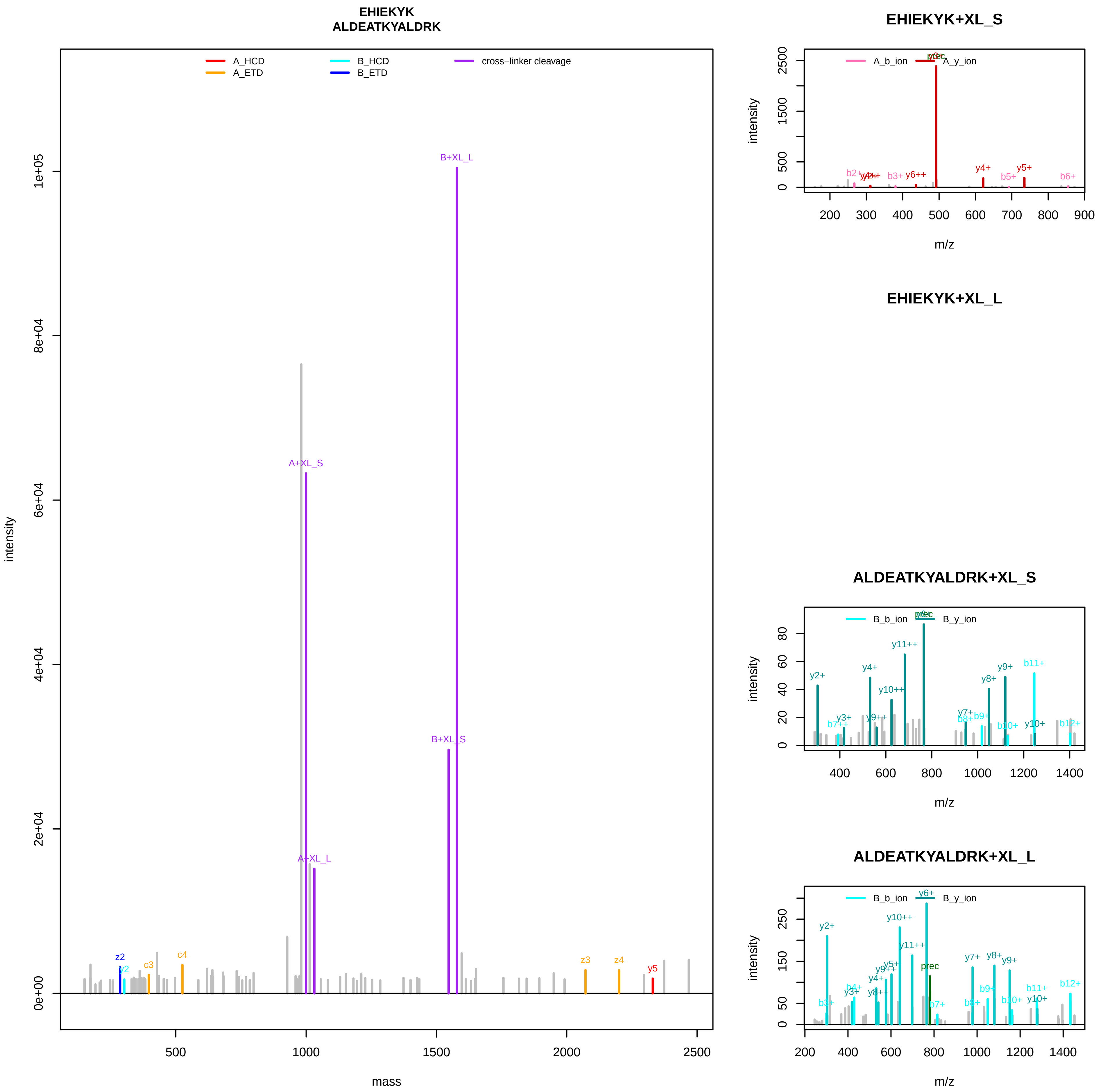

EHIEKYK
ALDEATKYALDRK
A_HCD
A_ETD
B_HCD
B_ETD
cross−linker cleavage
B+XL_L
A+XL_S
B+XL_S
A+XL_L
z2
y2
c3
c4
z3
z4
y5
intensity
mass
0e+00
2e+04
4e+04
6e+04
8e+04
1e+05
500
1000
1500
2000
2500
EHIEKYK+XL_S
A_b_ion
A_y_ion
prec
b2+
y2+
y4++
b3+
y6++
y4+
b5+
y5+
b6+
m/z
EHIEKYK+XL_L
ALDEATKYALDRK+XL_S
B_b_ion
B_y_ion
y2+
y3+
b7++
y4+
y9++
y10++
y11++
y6+
y7+
b8+
b9+
y8+
y9+
b10+
y10+
b11+
b12+
ALDEATKYALDRK+XL_L
b3+
b4+
y3+
y4+
y5+
y9++
y8++
y10++
y11++
prec
b7+
y7+
b8+
b9+
y8+
y9+
b10+
b11+
y10+
b12+
